# Improving CT prediction of treatment response in patients with metastatic colorectal carcinoma using statistical learning theory

**DOI:** 10.1186/1471-2164-11-S3-S15

**Published:** 2010-12-01

**Authors:** Walker H Land, Dan Margolis, Ronald Gottlieb, Elizabeth A Krupinski, Jack Y Yang

**Affiliations:** 1 Department of Bioengineering, Binghamton University, Binghamton, NY, 13903-6000, USA; 2 Department of Radiology, University of Arizona, Tucson, AZ 85724, USA; 3 Center for Research in Biological Systems, University of California at San Diego, La Jolla, California 92093-0043 USA; 4 Department of Radiation Oncology, Massachusetts General Hospital Cancer Center and Harvard Medical School, Boston, Massachusetts 02114 USA

## Abstract

**Background:**

Significant interest exists in establishing radiologic imaging as a valid biomarker for assessing the response of cancer to a variety of treatments. To address this problem, we have chosen to study patients with metastatic colorectal carcinoma to learn whether statistical learning theory can improve the performance of radiologists using CT in predicting patient treatment response to therapy compared with the more traditional RECIST (Response Evaluation Criteria in Solid Tumors) standard.

**Results:**

Predictions of survival after 8 months in 38 patients with metastatic colorectal carcinoma using the Support Vector Machine (SVM) technique improved 30% when using additional information compared to WHO (World Health Organization) or RECIST measurements alone. With both Logistic Regression (LR) and SVM, there was no significant difference in performance between WHO and RECIST. The SVM and LR techniques also demonstrated that one radiologist consistently outperformed another.

**Conclusions:**

This preliminary research study has demonstrated that SLT algorithms, properly used in a clinical setting, have the potential to address questions and criticisms associated with both RECIST and WHO scoring methods. We also propose that tumor heterogeneity, shape, etc. obtained from CT and/or MRI scans be added to the SLT feature vector for processing.

## Background

A major goal of this paper is to describe an ongoing research effort to ascertain the most important lesion features that change over time as rendered on Computed Tomography (CT), as well as other imaging modalities, using statistical learning theory (SLT) and complex adaptive system (CAS) paradigms, to reliably and reproducibly predict patient outcomes in response to targeted therapies for metastatic colorectal carcinoma. There is currently a great deal of interest in the establishment of radiologic imaging as a valid biomarker for assessing the response of cancer to a variety of treatments [1-8]. Imaging holds the promise of serving as an earlier, more accurate predictor of patient outcomes than serologic or clinical parameters [2,5,8-10]. CT is the most widely used imaging modality to assess the change in patient tumor burden using quantitative measures of tumor lesion volume such as the two dimensional WHO [11] or one dimensional criteria Response Evaluation Criteria in Solid Tumors (RECIST) [12] used to measure patient response.

Little work has been done in validating imaging as a surrogate endpoint for patient overall survival in response to the many new therapies that are being developed to treat advanced cancer in patients on defined protocols or for the vastly larger pool of patients having imaging used to assess their likely outcome in response to established therapies. To date there has been no mechanism to have radiologists consistently use reproducible metrics of tumor response validated by a high level of performance in predicting patient outcome at individual sites or collaborating sites regionally or nationally. Difficulties arise in the logistics of having radiologists reproducibly use similar terms and methods of measuring lesion change [13], and in relating imaging findings to patient outcome [14-16]. Most prior work has been directed at measuring lesion size on CT with RECIST and WHO (World Health Organization) measurements [11,12], and more recently with 3-D volumetric analyses [17-22] without considering how change in size relates to outcome. Other information contained on CT and magnetic resonance imaging (MRI) scans regarding lesion appearance (including perfusion, tracer activity, margins, and internal features) has not been addressed adequately [23-25].

RECIST 1.1, the current standard [26] used to evaluate treatment response for patients on new protocols for cancer, is a semi-quantitative scoring system which considers only existing lesion size change measurements and interval development of new lesions in placing patients into different response categories. Recently Positron Emission Tomography (PET) and PET fused with CT (PET/CT) have been used to assess cancer response, but the same issues of reproducibly relating and validating imaging findings with regard to patient outcome are present.

We have chosen to study patients with metastatic colorectal carcinoma for this pilot project to learn whether statistical learning theory can improve the performance of radiologists using CT in predicting patient treatment response to therapy compared with the more traditional RECIST standard. CT is currently the most commonly used imaging modality to evaluate response to treatment of a variety of solid tumors including colorectal carcinoma. Colorectal carcinoma arises from the epithelial lining cells of the large intestine. Systemic chemotherapy, with or without surgery and radiation becomes the treatment of choice for patients with metastatic disease. Survival in these patients is usually short, but therefore readily measurable as a marker for the success or failure of different treatments. Various new therapies are being developed to improve survival, which have assorted mechanisms of action including angiogenesis modulators and epidermal growth factor inhibitors, which can be evaluated by imaging biomarkers. More accurately predicting patient outcome in patients early in the course of therapy has the potential to accelerate drug development in phase II and phase III trials, improve patient survival, and avoid prolonged potentially toxic therapies in patients unlikely to do well. This research project considers CT and colon cancer but our methods of analyzing imaging results is readily applied to other modalities (e.g., PET/CT, MRI) and other types of malignancies.

RECIST (Response Evaluation Criteria in Solid Tumors) [14] and its predecessors, primarily the WHO method [11,27], define standard measurement methods for converting visual image observations into a quantitative and statistically tractable framework for measuring tumor size response to therapy. The RECIST criteria were modified in 2000 [12] to make measurement practices procedurally more consistent across multiple trials and accommodate improvements in CT and MRI scanners. Each method uses a pragmatically simplistic technique, which is dependent on observer judgment, to determine lesion boundaries. WHO defines its tumor measurement by summing a group of individual masses, each of which is assessed by the cross product of its greatest diameter and largest perpendicular diameter. RECIST uses a linear measure. RECIST was designed to be sufficiently aligned with past WHO practices such that no major discrepancy would occur in the partial response between the old and new guidelines, while specifying procedures on such items as the maximum number of solid tumor lesions that should be measured [28] and the maximum number of lesions measured in any one organ [29]. RECIST target lesions have to be acquired with image slice thicknesses no larger than one half the diameter of the measured lesion, which results in recommending that 10-mm objects be imaged with 5-mm image slice thicknesses, while limiting measurable target lesions to no smaller than 10mm except under special circumstances. After target lesions are measured using either single linear summation (RECIST) or the bilinear product approach (WHO), the results are subsequently assigned to response-defined categories of complete response (CR), partial response (PR), stable disease (SD), and progressive disease (PD). By an apparently subjective criteria, RECIST defined PR as a more than 30% linear decrease of the linear sums of the target lesions (thus, by extrapolation, implying a 65% volumetric decrease) and PD as a more than 20% increase (implying a 73% volumetric increase). This contrasts with WHO criteria, in which those boundaries were set volumetrically at 65% and 40%, respectively [30].

Because RECIST was framed in the context of individual slices (and generally axial in the case of CT) the research community is currently re-exploring the obvious gaps in both RECIST and WHO criteria, which are constrained by the limits of earlier technology. Little attention has been paid to acknowledging inter and intra observer variability. That is, the reader makes his/her measurements unassisted by anything other than the most rudimentary form of image-processing technology (often simply the use of electronic calipers on a workstation display), resulting in significant differences among readers and within single readers over time. Aside from suggesting the value of multiple independent observers, current approaches are hardly likely to improve decision making consistency on fuzzily bounded objects. Neither RECIST nor WHO provide especially rigorous guidance on the subject of observer variability aside from recommending review panels and independent observers. Disagreement among observers [30,31] has been noted to be as high as 15% to 40% in these contexts and may not be ideally remedied by consensus or tie preventing arrangements. In fact, there is little scientific literature that resolves the question of what would constitute a sufficient number of observers [32,33]. To minimize reader variation, three observers have generally been employed. This odd-numbered arrangement offers a pragmatic means of averaging data and avoiding a tie, but has no theoretical basis. Larger numbers of readers may permit some greater level of certainty but is impractical or unaffordable in a real-world context. Furthermore, providing only nominal guidance on slice thickness, RECIST does not address at any length image acquisition components that inevitably result in significant lesion contrast differences within and between studies.

However, RECIST has served a useful historic purpose in grouping image data into the four response classifications (CR, PR, SD, PD). Since diameter measurements are best determined on smoothly shaped, distinct tumor boundaries—an ideal circumstance encountered infrequently—measurement variability inherent in such judgments is not adequately reflected in the recorded data and is therefore a confounder that cannot be corrected systematically. Tumors with irregular or diffuse boundaries pose the most significant challenge to data extraction and are highly observer dependent.

***Despite these recognized limitations, studies continue to be directed towards refining the RECIST paradigm by minor, incremental modifications. This is a major reason for proposing this new SLT technology, which can take advantage of the new feature vector components resulting from these newer imaging modalities, such as CT and MRI.*** *Feature vector components are the elements of the input vector used by the statistical learning theory algorithms in arriving at an intelligent decision.*

Finally, the National Cancer Institute (National Institutes of Health, Bethesda, MD) Cancer Treatment Evaluation Program in its review of proposed trials acknowledges both WHO criteria and RECIST as useful for estimating tumor size response to therapies, but neither mandates nor requires either for use in its sponsored clinical trials.

Consequently, we propose to use Complex Adaptive Systems (CAS) and SLT to develop and test intelligent post processing software to address these problems. This software is designed, in part, to identify False Positive (FP) and False Negative (FN) errors. Patients that fall into the FN error group should have been treated but were not while the patients that fall into the FP error category needlessly suffer the morbidity of treatment as well as generating significant, unnecessary treatment cost. Consequently, *both* type I (FP) and type II (FN) errors are significant and should be minimized, which is possible with the proposed STL algorithms that adapt to the environment by using a feedback mechanism to develop intelligent emergent interpretation behavior by radiologists.

## Results

### RECIST, WHO individually and with additional information

Eight experiments were designed to measure the individual Performance SLT accuracy of RECIST and WHO as well as RECIST and WHO SLT improved performance with additional information. These experiments use the “one hold out” cross-validation technique because the validation sets for 5-fold cross-validation did not contain enough samples for statistically valid conclusions. Two SLT paradigms were used: a non-linear support vector machine (SVM) that employed a sigmoid mapping kernel, and a linear Logistics Regression (LR) approach.

The SVMs were manually trained by an iterative process rather than using an Evolutionary Programming (EP) approach, which if implemented may have resulted in an improved SVM system performance. The authors have developed and EP / Evolutionary Strategies (ES) SVM hybrid (EP/ES-SVM hybrid) that efficiently ascertains, by an optimal search mechanism, the optimal mapping kernel parameters used in mapping the feature data from the non-linear input space to the linear feature space. This hybrid was not used in this analysis because the software package used to configure the dataset for experiments, perform the SVM and LR techniques, and then run the Receiver Operator Characteristic (ROC) analysis did not contain the ability to use these advanced EP/ES-SVM theories. Rather, an iterative process of manually changing the SVM parameters in small steps was employed. Orange, the software package used, acts as a wrapper for the well known LIB SVM libraries, but does not offer any optimization algorithms. This **software** package requires the user to ascertain, by experiment, the mapping kernel parameters which result in the best possible performance.

The group evaluated included 38 patients with metastatic colon cancer treated with a last line therapy (cetuximab and irinotecan), who had a median survival of 8 months. Survival was used as the gold standard diagnosis for Measures of Performance (MOP). A good response was considered survival beyond 8 months (TP) and a poor response was considered death at or prior to 8 months (TN). The experiments had either 114, 38, or 31 samples, which were representative of the 114 lesions, 38 patients who had at least one lesion, and 31 patients who had multiple lesions. When only one or two lesions per patient are used, lesions were selected at random. The total feature vector length contained 35 components, where each experiment utilized subset elements of this feature vector. Table 1 depicts the information content for these eight experiments: four for RECIST and four for WHO, and delineates the number and type of feature(s) used as well as the sample size.

**Table 1:** List of Experiments for Lesion Measurement Standards

Experiment #	Features	# of features	# of samples
1	RECIST Size Change or WHO Size Change only for 1 Lesion by Both Observers	4	38

2	# of Lesions per Patient, RECIST Size Change or WHO Size Change only, and Visual Size Change by Both Observers	9	114

3	# of Lesions per Patient, New Lesions, RECIST Size Change or WHO Size Change only, and Visual Size Change for 2 Lesions by Both Observers	17	31

4	# of Lesions per Patient, New Lesions, RECIST Size Change or WHO Size Change, and Visual Size Change for 1 Lesion by Both Observers	11	38

Experimental designs to ascertain performance accuracy for RECIST and WHO

**Figure 1:**
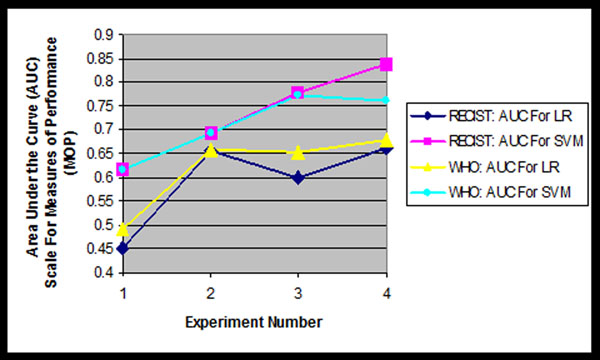
**Results of Lesion Measurement Standards Experiments** RECIST and WHO Performance Resulting From Non-Linear Support Vector Machine (SVM) and Linear Logistics Regression (LR) Processing. We propose establishing and quantifying continuous MOPs to replace the four discrete RECIST and WHO MOPs currently in use

Area under the ROC curve (AUC) performance results for the four RECIST only and the four WHO only experiments are depicted in figure 1. Figure 1 contains 4 curves. Curves 1 and 2 are AUC SVM and LR values for RECIST only, while curves 3 and 4 are the SVM and LR results for WHO only. In general, this set of studies showed the following:

• SVM performance for both RECIST and WHO are better than the LR performance for all experiments (compare curves 1 and 2 to curves 3 and 4). This is an expected result because the SVM paradigm captures predictive and discriminating information “hidden” in the non-linear regions of the input space, where the LR paradigm, because of its linear processing only, cannot “see” this discriminating information.

• RECIST performed slightly better than WHO for experiments 1, 2 and 3, but out performed WHO in Experiment 4, when using SVM processing (compare curves 2 and 4). *{Note: Remember that two sets of 4 experiments were performed: 4 for RECIST and 4 for WHO}.This* means that including more patients (diversity) with only one lesion has more predictive power than including less patients with 2 or more lesions, at least for this population. This preliminary result may disagree with intuition, which says that more lesions/patient provides more information. However, with these non-linear algorithms perhaps the manner in which the feature vector components interact and their information diversity may be more significant than the number of lesions/patient processed. This is an area for further research study using experimental sensitivities.

• Both RECIST and WHO alone performed equally, but with a fair to poor AUC of 0.61 using the non-linear kernelized SVM paradigm. (See Experiment 1 for curves 1 and 3).

• Logistics Regression (LR) performed worse, especially with RECIST, when using 2 lesions per patient compared to using just 1 lesion. This may be because more patients complicated the structure of the input space, thereby making it more non-linear because of coupling effects. Consequently, the linear RECIST measures cannot capture the information in these non-linear regions, but WHO can with a higher resultant AUC of ~0.65. This may be because WHO defines its tumor measurement by summing a group of individual masses; each lesion is assessed by the cross product of its greatest diameter and its largest perpendicular bisector, a mathematical operation that contains a non-linear component. This is also a topic for further investigation.

• Experiment 2, where all lesions are processed independent of patient, provides the least improvement between LR and SVM processing when compared to the baseline Experiment 1 (that uses RECIST only and WHO only), as well as when using additional information (See Experiments 3 and 4). That is, SVM processing for Experiment 2 increased the AUC for both RECIST and WHO performance from ~0.65 to ~0.69 (a ~7% improvement). However, the SVM processing for Experiments 3 and 4 increased performance, when compared to RECIST and WHO alone, by an average of ~25% (increase from ~0.62 to ~0.78 AUC average improvement) for Experiment 3 and an average improvement of ~23% (increase from ~0.66 to ~0.81 AUC average improvement) for Experiment 4 (see average of experimental values for curves 1 and 3.) This is also a topic for further research.

• Note the negatively correlated LR values for Experiment 1. This result occurred for both RECIST and WHO, and is again a reflection of the fact that linear processing techniques cannot capture information “hiding” in the non-linear region of the solution space. Note that the non-linear processing provided by the SVM for both RECIST and WHO using only these feature inputs resulted in a ~0.61 AUC. This result says that, when compared to Experiments 2 through 4, both the basic RECIST and WHO benefited from additional information added in the input feature vector. This result implies adding imaging components to the feature vector should also increase performance and we will quantify this measured improvement.

• Because of the marginal SVM and LR results from Experiment 1, we are actively investigating (and developing) other MOPs which might be more sensitive to RECIST and WHO measurements, when supplemented with additional information, as was done in Experiments 3 and 4. This is an ongoing research effort.

• We have the capability to evaluate the efficacy of drug treatment related to vascularity and lesion enhancement. However, initial lesion enhancement experiments did not improve performance for the cases studied.

• In summary, we suggest that this preliminary research study has demonstrated that these SLT algorithms, properly designed, tested, evaluated and properly used with a computer in a clinical setting, has the potential to address those questions discussed in section 1.1 as well as those problems delineated in section 1.2 . We also suggest that tumor heterogeneity and shape, etc, obtained from CT and/or MRI scans, be added to the feature vector for processing. This will be a simple process when the data is available.

### Observer variability

We have previously discussed (see Background), lack of rigorous guidance for RECIST and WHO regarding observer variability except from recommending panels and independent observers. We hypothesize that both SLT and CAS can help in reducing observer interpretive variability by: (1) using automated intelligent computer processing, and (2) training observers using SLT paradigm outputs. That is, CAS and SLT do not make subjective judgments: they adapt to the environment by developing emergent behavior using only factual information contained in the feature vector and will adapt differently when this information content is altered in some way. They also recognize and better adapt to the more accurate information content contained in the feature vector. This is illustrated by the three experiments described in table 2, whose content is similar to that described in table 1.

**Table 2:** List of Experiments for Observer Variability

Experiment #	Features	# of features	# of samples
1	# of Lesions per Patient, RECIST Size Change, WHO Size Change, and Visual Size Change for 1 Lesion by Observer 1 or Observer 2 only	7	114

2	# of Lesions per Patient, RECIST Size Change, WHO Size Change, and Visual Size Change for 2 Lesions by Observer 1 or Observer 2 only	13	31

3	# of Lesions per Patient, New Lesions, RECIST Size Change, WHO Size Change, and Visual Size Change for 1 Lesion by Observer 1 or Observer 2 only	8	38

Experimental design to ascertain observer variability

**Figure 2:**
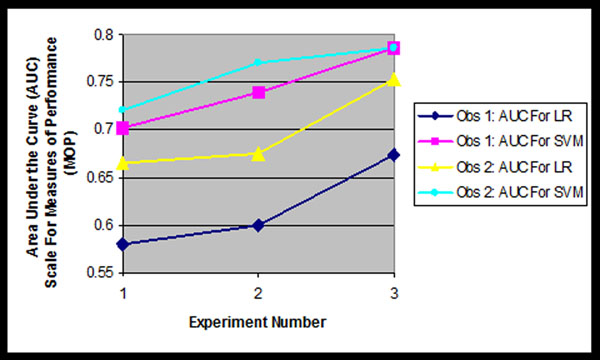
**Results of Observer Variability Experiments** Observer 2 is the most accurate reader. We propose to train Observer 1 (and other observers) using uniquely designed sensitivity experiments process by our SLT algorithms.

Figure 2 shows both SVM and LR SLT results for table 2 experiments for two observers, using the same data set described above. Two results are immediately clear: (1) Observer 2 has the best MOPs for predicting patient survival as expected being the more experience observer (three years of training compared to only one for Observer 1), and (2) SVM processing provided more accurate AUC results than LR, which is an expected result. However, another observation is that the SVM (and other) results can possibly be used to improve Observer 1’s performance by designing a set of sensitivity experiments to establish which inaccurately “read” feature values are most significantly contributing to performance denegation, and train Observer 1 using this information.

Finally, this set of observer variability experiments shows that Experiment 3 provides the most accurate results, which is consistent with the AUC results found in the previous set of experiments.

## Conclusions

Eight experiments were designed to measure the individual performance SLT accuracy of RECIST and WHO as well as RECIST and WHO SLT improved performance with additional information. These experiments used the “one hold out” cross-validation technique because the validation sets for 5-fold cross-validation did not contain enough samples for statistically valid conclusions. Two SLT paradigms were used: a non-linear support vector machine (SVM) that employed a sigmoid mapping kernel and a linear Logistics Regression (LR) approach. The group evaluated included 38 patients with metastatic colon cancer treated with a last line therapy (cetuximab and irinotecan), who had a median survival of 8 months. Survival was used as the gold standard diagnosis for MOP. A good response was considered survival beyond 8 months (TP) and a poor response was considered death at or prior to 8 months (TN). The experiments performed resulted in the following general behavior:

• SVM performance for both RECIST and WHO are better than the LR performance for all experiments. This is an expected result because the SVM paradigm captures predictive and discriminating information “hidden” in the non-linear regions of the input space, where the LR paradigm, because of its linear processing only, cannot “see” this discriminating information.

• We suggest that this preliminary research study has demonstrated that these SLT algorithms, properly designed, tested, evaluated and properly used with a computer in a clinical setting, has the potential to address those questions discussed in section 1.1 as well as those problems delineated in section 1.2 . We also suggest that tumor heterogeneity and shape, etc, obtained from CT and/or MRI scans, be added to the feature vector for processing.

• Two results for the observer variability experiments were immediately clear: (1) Observer 2 has the best MOPs for predicting patient survival as expected being the more experience observer (three years of training compared to only one for Observer 1), and (2) SVM processing provided more accurate AUC results than LR, which is an expected result. However, another observation is that the SVM (and other) results can possibly be used to improve Observer 1’s performance by designing a set of sensitivity experiments to establish which inaccurately “read” feature values are most significantly contributing to performance denegation, and train Observer 1 using this information.

• Finally, we have the capability to evaluate the efficacy of drug treatment related to vascularity and lesion enhancement. However, initial lesion enhancement experiments did not improve performance for the cases studied.

## Methods

### Summary of logistic regression, odds ratio and ROC curves

Ordinary regression deals with finding a function that relates a continuous outcome variable (dependent variable *y*) to one or more predictors (independent variables *x*_1_, *x*_2_, etc.). Simple linear regression assumes a function of the form: *y =* c_0_ + c_1_*x*_1_ + c_2_*x*_2_ +Â…… and finds the values of c_0_, c_1_, c_2_, etc. Logistic regression is a variation of ordinary regression, useful when the observed outcome is restricted to a dichotomous output, which usually represents the occurrence or non-occurrence of some outcome event, (usually coded as 1 or 0, respectively). It produces a result that predicts the probability of the occurrence as a function of the independent variables. Logistic regression fits a special sigmoidal curve by taking the linear regression, which could produce any *y*-value in the range [-∞, +∞] and transforming it with the function: *p =* Exp(*y*) / (1 + Exp(*y*)), which produces *p*-values between 0 (as *y* approaches minus infinity) and 1 (as *y* approaches plus infinity). Consequently, this now becomes a special kind of regression, which also produces Odds Ratios (OR) associated with each predictor value. The ***odds*** of an event are defined as the probability of the outcome event occurring divided by the probability of the event not occurring. The **odds ratio** for a predictor tells the relative amount by which the odds of the outcome increase (OR greater than 1.0) or decrease (OR less than 1.0) when the value of the predictor is increased by 1.0 units.

However, a problem exists with using OR alone [34]. The accuracy or validity of a binary marker for classifying persons is better summarized in a case-control study by reporting its true-positive fraction (TPF, also known as sensitivity) and its false-positive fraction (FPF, also known as 1-specificity) These are defined as follows: TPF = Prob.[marker positive | outcome positive] and FPF = Prob.[marker positive | outcome negative]. Because there are two types of errors (misclassifying positives and misclassifying negatives), the study results should reflect both of these errors. A perfect marker will have TPF = 1 and FPF = 0. Obviously, to have confidence in the prediction that a marker makes, TPF and FPF should be close to these ideal values. The general public often expects that a marker offer reasonably accurate classification and confident prediction. However, a marker can be useful even if FPF and TPF are less than ideal. The criteria by which the marker is judged useful depend entirely on the context in which it is to be used. For example, a marker for screening a healthy population for cancer needs to have an extremely low FPF because workup procedures such as biopsy that follow a positive screening test are generally invasive and expensive. Given that cancer is a rare disease in the population tested, even a low FPF will result in huge numbers of people undergoing unnecessary, costly procedures. The odds ratio can be written as a simple function of (FPF, TPF) [35,36]:

OR = (TPF / (1 – TPF))*((1 – FPF) / FPF)

The difficulty with this definition is that it depends upon a specific threshold setting (as specific values of TPF and FPF are required), and consequently the odds ratio is a simple scalar measure of association between marker and outcome. Therefore, OR values do not characterize the discrimination between cases and controls that can be achieved by a marker since an infinite number of pairs of TPFs and FPFs are consistent with a particular odds ratio value (because TPF is a continuous mapping of FPF for a specific value of OR).

Consider the two density functions of a particular test in two populations; where one population has a disease (or some other meaning) and the other population does not have a disease (or some other meaning). One rarely observes a perfect separation between these two density functions, but rather an overlap in the distribution of the test results, which give rise to both false positive and false negative errors. ROC curves are one way to measure the amount of overlap between the probability density functions, and are built from these two density functions by first constructing two functions, where the first graph is hit rate (or sensitivity) as a function of threshold setting, while the second graph is the false alarm rate (or 1 –specificity) as a function of threshold setting. Then the hit rate is plotted as a function of the false alarm rate using the threshold value as the parameter. Finally, the AUC follows by numerical integration, which is bounded by 0 ≤ AUC ≤ 1 and has the following meaning: 0.5, which is equivalent to random guessing, and increasing to 1 for a perfect system. Therefore, the AUC (sometimes called the AZ value) may be interpreted as the average system performance as measured over all threshold settings. Consequently, the ROC curve is the natural generalization of (FPF, TPF) to accommodate settings in which the marker is continuous. It describes the whole set of potential (FPF, TPF) combinations possible with positivity criteria based on the marker. Changing the units in which the marker is measured has no impact on its ROC curve in contrast to logistic regression models in which, as noted above, the odds ratio must be interpreted according to a unit increase in the value of *X.* Moreover, ROC curves provide a natural common scale for comparing different markers even when they are measured in completely different units. In contrast, because odds ratios are interpreted per unit increase in the marker, odds ratios for two markers may not be comparable. This is why we convert the logistic regression probability density functions into ROC curves for this paper.

#### Summary of logistic regression

Logistic regression is a multivariate extension of linear regression, used for predicting a dichotomous outcome, usually non-occurrence or occurrence of an event represented by 0 or 1 respectively [37].

##### Logit transformation

As Y approaches 1 or 0, a one unit change in *X* should have a smaller effect then when *Y* is around 0.5. The varying change in *Y* based on the magnitude of *X* is accomplished in logistic regression with the Logit transformation. Let *P_i_* be the probability of event *E* occurring at *X_i_.* Then the odds of *E* occurring at *Xi* are *P_i_* / (1 - *P_i_* ). The Logit, or logged odds, is the natural logarithm of the odds *ln*(*P_i_* / (1-*P_i_*)). The relationship of *X* to the Logit, or logged odds, is given by:

*b*_0_ + *b*_1_*X_i_* = *ln*(*P_i_* / (1 - *P_i_*)),

where *b*_0_ is the intercept and *b*_1_ is the coefficient, such that each unit change in *X* results in a *b_1_* change in the log odds. Consequently, it follows from the above formula that *X* is related to the odds by:

*P_i_* /(1 – *P_i_*) = *e*^*b*_0_+*b*_1_*X_i_*^

The multivariate formula for the logged odds would then be

*P_i_* /(1 – *P_i_*) = *e*^*b*_0_+*b*_1_*X*_*i*1_+*b*_2_*X*_*i*2_+…*b_n_X_in_*^

The probability may be calculated from *X_i_*, the intercept and coefficients, as described in the expression below:

*P_i_* = *e*^*b*_0_+*b*_1_*X*_*i*1_+*b*_2_*X*_*i*2_+…*b_n_X_in_*^ / (1 + *e*^*b*_0_+*b*_1_*X*_*i*1_+*b*_2_*X*_*i*2_+…*b_n_X_in_*^)

These density functions are also used to develop the logistic regression ROC curves, where this development will be subsequently described.

##### Log likelihood

The log likelihood is used in the selection of the coefficients as well as a test of model fitness. The likelihood of a model is Π{*P_i_^Y_i_^* * (1 – *P_i_*)^1 –*Y_i_*^} such that *P_i_* is the calculated probability of the result being 1, and *Y_i_* is the actual result for sample *i.* Note that the likelihood is 1 for a perfect model where *P_i_ = Y_i_* such that *Y* is in {0, 1}. To avoid minute values, logistic regression uses the logged likelihood or

∑ {(Y_i_* ln(*P_i_*)) + (1 – *Y_i_*) * ln(1 – *P_i_*)}

As the likelihood function approaches 1, the log likelihood approaches 0. The smaller the results of the likelihood function, the further negative the log likelihood. The closer the likelihood is to 1, the more representative the coefficients and intercept are of producing the observed results.

##### Intercept and coefficients

The intercept and coefficients are chosen to maximize the logged likelihood. The intercept is first set to the base logged likelihood. The base log likelihood is calculated with all coefficients set to zero and *P_i_* set to the probability of the outcome in the sample set. The intercept and coefficients are then modified in an iterative fashion using the Newton-Raphson method to achieve the maximum logged likelihood. The solution is said to converge when the change in the log likelihood is less than 10^-8^ from the previous iteration.

##### Base log likelihood

The base log likelihood is the value where the model performs no better than chance. The further the final log likelihood from the base log likelihood the better the model fit. This distance is then multiplied by -2 giving a chi-square value with the degrees of freedom equal to the number of independent variables.

##### Converting PDFs into ROC curves

We previously stated that the multivariate density functions given below were used to formulate the ROC curves.

*P_i_* = *e*^*b*_0_+*b*_1_*X*_*i*1_+*b*_2_*X*_*i*2_+…*b_n_X_in_*^ / (1 + *e*^*b*_0_+*b*_1_*X*_*i*1_+*b*_2_*X*_*i*2_+…*b_n_X_in_*^)

ROC curves developed directly from probability density functions eliminate the problems previously discussed using odds ratios as well as allow an “apples-to-apples” comparison with ROC results developed by more standard approaches. The process is as follows. From either the null or alternate hypothesis density function, compute the hit rate and false alarm rate as a function of the threshold setting and from these two functions construct the hit rate as a function of the false alarm rate using the same activation threshold parameter. The area under the curve (AUC), or AZ value, may then be computed by a trapezoidal integration.

### Summary of support vector machines

Support vector machines (SVMs), as developed by Vapnik [38-42] are briefly summarized in this section. This discussion is included to provide the theoretical explanation for why it is possible to train SVMs to a global minimum, and thereby provide better performance accuracy, when compared to three layer artificial neural network performance trained by back propagation. Assume there exist N observations from a data set. Each observation (or training example) consists of a vector x_i_ containing the input pattern and a corresponding known classification y_i_. The objective of the learning machine would be to formulate a mapping x_i_ → y_i_. Now consider a set of functions f(x, α) with adjustable parameters α, that define a set of possible mappings x → f(x, α). Here, x is given and α is chosen. In the case of a traditional neural network of fixed architecture, the α values would correspond to the weights and biases. The quantity R(α), known as the expected (or true) risk, associated with learning machines is defined as:

where, p(x, *y*) is an unknown probability density function from which the examples were drawn. This risk function is the expected (or true) value of the test (or validation) error for a trained learning machine. It may be shown that the best possible generalization ability of a learning machine is achieved by minimizing *R*(α)*,* the expected (or true) risk. This generalization bound, for binary classification, holds with the probability of at least 1 - η (0 ≤ η ≤ 1) for all approximating functions that minimize the expected (or true) risk.

The first term on the right hand side of the above equation is known as the “empirical risk”, where the empirical risk *R*_emp_(α) is expressed by:

This function is a measure of the error rate for the training set for a fixed, finite number of observations. This value is fixed for a particular choice of α and a given training set (x*_i_*, *y_i_).* The second term in the above expression is the “Vapnik-Chervonenkis (VC) confidence interval.” This term is a function of the number of training samples *N*, the probability value η and the VC dimension *h.* The VC dimension is the *maximum* number of training cases that can be learned by a learning machine without error for *all* possible labeling of the classification functions *f*(x, α), and is, therefore, a measure of the capacity of the learning machine. In traditional neural network implementations, the confidence interval is fixed by choosing a network architecture *a priori.* The function is minimized by generally obtaining a local minimum from minimizing the empirical risk through adjustment of weights and biases. Consequently, neural networks are trained based on the empirical risk minimization (ERM) principle. In an SVM design and implementation, not only is the empirical risk minimized, the VC confidence interval also is minimized by using the principles of structural risk minimization (SRM). Therefore, SVM implementations simultaneously minimize the empirical risk as well as the risk associated with the VC confidence interval, as defined in the above expression. The above expression also shows that as *N* → ∞, the empirical risk approaches the true risk because the VC confidence interval risk approaches zero. The reader may recall that obtaining larger and larger sets of valid training data would sometimes produce (with a great deal of training experience) a better performing neural network, which resulted from classical training methods. This restriction is not incumbent on the SRM principle and is the fundamental difference between training neural networks and training SVMs. Finally, because SVMs minimize the true risk, they provide, when properly trained, a global minimum.

### Summary of data set feature components and feature vector construction

Seventy five potential features were available for use in the experiments: (1) those that could be used for all experiments, (2) those that could be used only for experiments where the lesions were analyzed individually, and (3) those that could be used only for experiments where the patient was being analyzed as a whole. The features comprising the data set were empirically determined and organized into a format suitable for statistical learning theory processing. While all the patients had at least one lesion, only four had the full six lesions possible in the feature vector. Table 3,4,5 depict the name in the database of each feature, and a detailed description of what that feature represents.

**Table 3:** Feature Vector for Lesions or Patients

For Either Patient or Lesion Based Experiments	
*The following features could be used in both analyzing a patient as a whole or the lesions individually:*	

Num_Lesions	Number of Lesions per Patient

T_ Overall	Overall Visual Tumor Burden Change – Observer 1

C_Overall	Overall Visual Tumor Burden Change – Observer 1

T-NewL	Patient has New Lesions – Observer 1

C-NewL	Patient has New Lesions – Observer 2

Features that could be used for either lesion or patient based experiments

**Table 4:** Feature Vector for Lesions Only

Lesion Based Experiments	
*When lesions were analyzed individually, each lesion had the following:*	

T_ RECIST1	Baseline RECIST Size Change – Observer 1

C_RECIST1	Baseline RECIST Size Change – Observer 2

T _WHO1	Baseline WHO Size Change – Observer 1

C _WHO1	Baseline WHO Size Change – Observer 2

T_ RECIST2	Follow up CT RECIST Size Change – Observer 1

C_RECIST2	Follow up RECIST Size Change – Observer 2

T _WHO2	Follow up WHO Size Change – Observer 1

C _WHO2	Follow up WHO Size Change – Observer 2

T-Target	Visual Tumor Change in Target Lesion – Observer 1

C-Target	Visual Tumor Change in Target Lesion – Observer 2

Features that could be used for only individual lesion based experiments

**Table 5:** Feature Vector for Patients Only

Patient Based Experiments	
*Patients had from 1 to 6 lesions. Each lesion had the following features: (# denotes lesion number)*	

#-T_ RECIST 1	Baseline RECIST Size Change – Observer 1

#-C RECIST1	Baseline RECIST Size Change – Observer 2

#-T _WHO1	Baseline WHO Size Change – Observer 1

#-C _WHO1	Baseline WHO Size Change – Observer 2

#-T_ RECIST2	Follow up RECIST Size Change – Observer 1

#-C_RECIST2	Follow up RECIST Size Change – Observer 2

#-T _WHO2	Follow up WHO Size Change – Observer 1

#-C _WHO2	Follow up WHO Size Change – Observer 2

#-T-Target	Visual Tumor Change in Target Lesion – Observer 1

#-C-Target	Visual Tumor Change in Target Lesion – Observer 2

Features that could be used for only patient based experiments. No more than two lesions were selected per patient in any experiment

### Applying statistical learning theory to access change in tumor size in response to therapy using the basic RECIST and WHO criteria with additional information

This preliminary research study documents SLT performance for the following experiments: (1) RECIST and WHO individually, (2) RECIST and WHO with additional information, and (3) observer variability using RECIST and WHO. These SLT experiments use a basic population MOP, which is the area under the ROC. AUC is a quantitative population MOP as our current objective is to establish the performance for a group of patients, and later extend our SLT algorithms to individual patient risk assessment by using, for example, epidemiological MOPs such as Odds Ratios, Relative and Absolute Risk.

**Figure 3:**
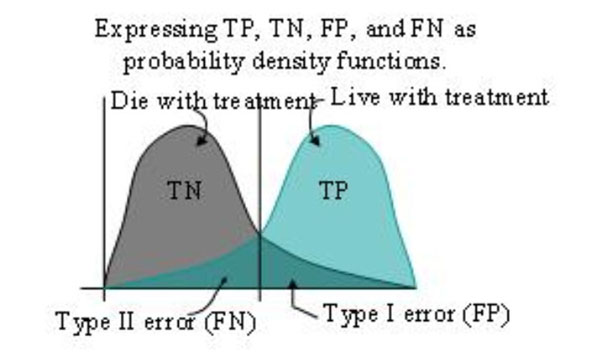
Probability Density Functions

**Figure 4:**
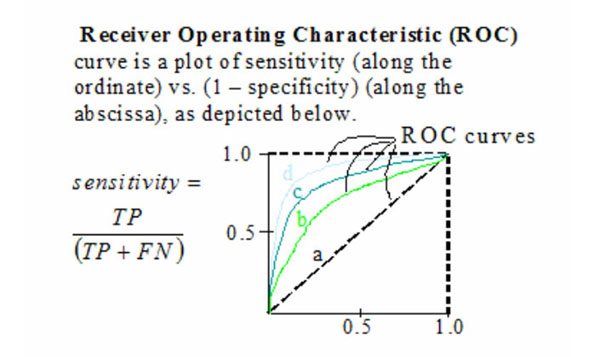
ROC Curve

Figures 3 and 4 depict the definition of the density functions as well as the resultant form of the ROC curve. This AUC could be used to supplement the currently used discrete RECIST and WHO categories of complete response (CR), partial response (PR), stable disease (SD), and progressive disease (PD).

TP, TN, FN, and FP are defined in table 6. Patients that fall into the FP error category needlessly suffer the morbidity of treatment as well as generating significant, unnecessary treatment cost while the patients that fall into the FN error category should have been treated but were not. Consequently, *both* type I (FP) and type II (FN) errors are significant and should be minimized, which is possible with SLT algorithms that adapt to the environment by using a feedback mechanism and thereby intelligently develop emergent behavior.

**Table 6:** Contingency Matrix for Patient Outcome Prediction

	System Diagnosis	
		+	-

Gold Standard Diagnosis	+	TP (true positive) Patients that live with treatment after 8 month period as expected	FN (false negative) Patients that will live after 8 months with treatment but are expected to die

	-	FP (false positive) Patients that die before 8 months with treatment but are expected to live	TN (true negative) Patients that die with treatment before 8 month period as expected

Contingency Matrix identifying statistical decision density function processing definitions and the resultant clinical errors possible. The Gold Standard is the correct outcome and the System Diagnosis is the prediction of outcome made

## Competing interests

The authors declare that they have no competing interests.

## Authors’ contributions

WHL developed new and refined existing (as appropriate) statistical learning theory (SLT) and complex adaptive systems (CAS) approaches employed in this paper. WHL, DM, RG were involved in the experimental design used to ascertain the efficacy of these SLT algorithms in assessing the treatment of metastatic colorectal carcinoma. EAK and JYY were involved in the results analysis and provided many useful scientific insights. All co-authors participated in the final analysis and reviews of the results of these experiments and agreed on the content of the paper.
